# The Effect of *Lycium barbarum* Polysaccharides on Pyroptosis-Associated Amyloid β_1-40_ Oligomers-Induced Adult Retinal Pigment Epithelium 19 Cell Damage

**DOI:** 10.3390/ijms21134658

**Published:** 2020-06-30

**Authors:** Ming Yang, Kwok-Fai So, Amy Cheuk Yin Lo, Wai Ching Lam

**Affiliations:** 1 Department of Ophthalmology, Li Ka Shing Faculty of Medicine, The University of Hong Kong, Hong Kong, China; hrmeym@hku.hk (M.Y.); hrmaskf@hku.hk (K.-F.S.); 2 State Key Laboratory of Brain and Cognitive Sciences, The University of Hong Kong, Hong Kong, China

**Keywords:** cell death, drusen, eye disease, retina, traditional Chinese medicine (TCM)

## Abstract

Age-related macular degeneration (AMD) is a sight-threatening disease with limited treatment options. We investigated whether amyloid β_1-40_ (Aβ_1-40_) could cause pyroptosis and evaluated the effects of *Lycium barbarum* polysaccharides (LBP) on Aβ_1-40_ oligomers-induced retinal pigment epithelium 19 (ARPE-19) damage, which is an in vitro AMD model. Aβ_1-40_ oligomers verified by Western blot were added to ARPE-19 cells with or without 24 h LBP treatment. Aβ_1-40_ oligomers significantly decreased ARPE-19 cell viability with obvious morphological changes under light microscopy. SEM revealed swollen cells with a bubbling appearance and ruptured cell membrane, which are morphological characteristics of pyroptosis. ELISA results showed increased expression of IL-1β and IL-18, which are the final products of pyroptosis. LBP administration for 24 h had no toxic effects on ARPE-19 cells and improved cell viability and morphology while disrupting Aβ_1-40_ oligomerization in a dose-dependent manner. Furthermore, Aβ_1-40_ oligomers up-regulated the cellular immunoreactivity of pyroptosis markers including NOD-like receptors protein 3 (NLRP3), caspase-1, and membrane N-terminal cleavage product of GSDMD (GSDMD-N), which could be reversed by LBP treatment. Taken together, this study showed that LBP effectively protects the Aβ_1-40_ oligomers-induced pyroptotic ARPE-19 cell damages by its anti-Aβ_1-40_ oligomerization properties and its anti-pyroptotic effects.

## 1. Introduction

Age-related macular degeneration (AMD) is a leading cause of blindness and irreversible visual impairment in the rising aging population. It is estimated that the number of AMD patients worldwide will grow from 196 million in 2020 to 288 million by 2050 [1]. Early intervention of AMD is essential for preventing the aggravation of the disease [2,3,4]. The current therapies for the early stage of AMD are not satisfactory [5].

In the early stage of AMD, drusen formation and accumulation, and retinal pigment epithelium (RPE) cell damage are two prominent pathological features. Amyloid β_1-40_ (Aβ_1-40_) is often found in drusen, while amyloid β_1-42_ (Aβ_1-42_) is the major constituent of plaques, which is a prominent feature in the brains of Alzheimer’s disease patients [6,7]. Indeed, Aβ_1-40_ oligomers have been shown to be the predominant component of drusen in the postmortem eye of a 72-year-old AMD patient [8]. Previous studies showed that exposure to Aβ_1-40_ oligomers resulted in a decrease in RPE cell viability [9,10]. However, the intracellular mechanism(s) of RPE cell damage induced by Aβ_1-40_ oligomers is unclear. A better understanding of this process will provide insights into potential treatment strategies for AMD.

Pyroptosis is a programmed cell death pathway. It starts with the activation of the inflammasome, including NOD-like receptors protein 3 (NLRP3s), the adaptor protein ASC (Apoptosis-associated speck-like protein containing a caspase recruitment domain), and the precursor of caspase-1 (pro-caspase-1) [11,12,13]. Subsequently, procaspase-1 is cleaved to form caspase-1. On one hand, caspase-1 participates in innate immunity and it activates the pro-inflammatory cytokines such as pro-interleukin-1β (pro-IL-1β) and pro-interleukin-18 (pro-IL-18), resulting in the secretion of IL-1β and IL-18 to trigger an inflammatory response [14]. On the other hand, caspase-1 cleaves GSDMD, which is one of the Gasdermin family members [12,15]. The N-terminal cleavage product of GSDMD (GSDMD-N) causes extensive cell perforation by insertion into the lipid bilayer of the membrane [16]. This is followed by the release of cellular content, causing cell swelling and lysis [17,18,19]. Early studies showed that the inflammasome was activated in geographic atrophy or neovascular AMD patients [20]. However, whether pyroptosis exists in the AMD patients has not yet been reported.

Using the all-trans retinal model for AMD, a study found that caspase-3/gasdermin E-mediated pyroptosis was activated [21]. Nevertheless, whether the drusen component (Aβ_1-40_) could cause pyroptosis is not clear.

*Lycium barbarum* polysaccharides (LBP) is a key active component of Goji berry (also called Wolfberry or Gouqizi), which has been widely used as a Traditional Chinese Medicine (TCM) for more than 2500 years in Asia with no reported toxic effects. LBP is composed of polysaccharides, monosaccharides, uronic acid, acidic heteropolysaccharides, polypeptides, and proteins [22,23]. Moreover, there have been evidence that LBP has potential therapeutic effects on various eye diseases, including glaucoma [24], retinitis pigmentosa [25], diabetic retinopathy [26], and retinal ischemia–reperfusion injury [27] by its anti-oxidative, anti-inflammatory, and anti-apoptotic properties. However, little research on LBP addressed its effects on AMD using either in vivo or in vitro model.

Therefore, the present research is aimed to address three questions: (1) what are the mechanism(s) and the underlying signaling pathways for Aβ_1-40_ oligomers-induced RPE cell damage; (2) does LBP have a protective effect on RPE cell damage caused by Aβ_1-40_ oligomers; and (3) can LBP treatment regulate these signaling pathways.

## 2. Results

### 2.1. Aβ_1-40_ Oligomers Were Generated by Oligomerization Assay

**Figure 1:**
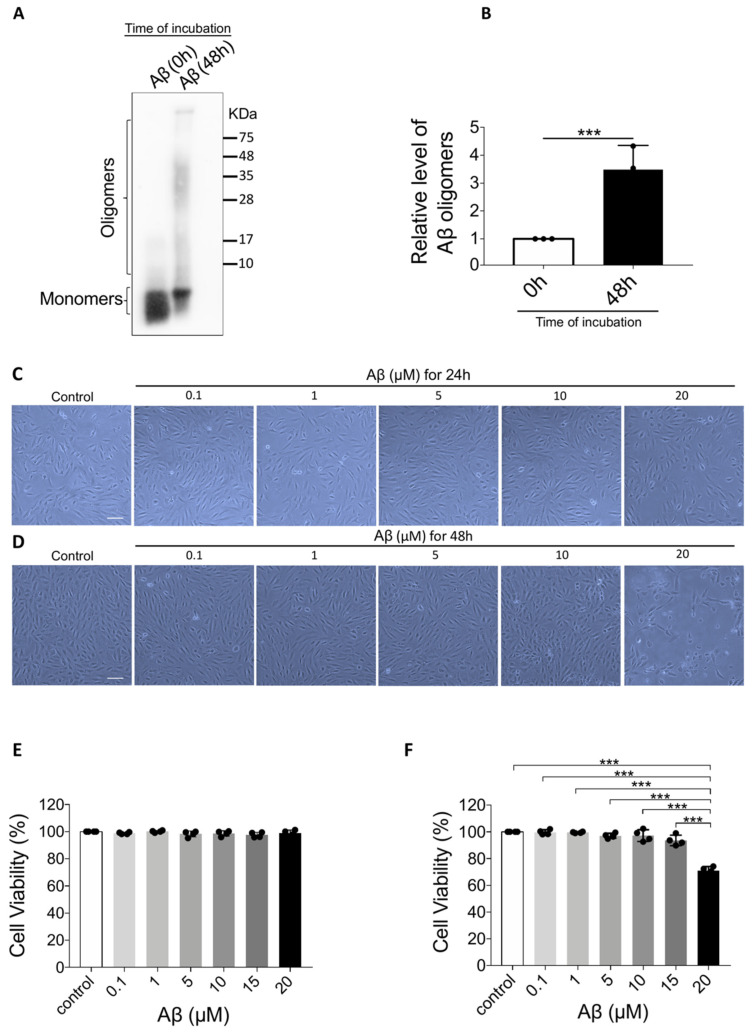
Confirmation of Aβ_1-40_ oligomerization and the effect of Aβ_1-40_ oligomers on Aβ_1-40_ oligomers-induced retinal pigment epithelium 19 (ARPE-19) cell viability. (**A**) Representative Western blot showing Aβ_1-40_ monomers and oligomers at 0 h and 48 h after oligomerization. (**B**) Semi-quantification of Aβ_1-40_ oligomers showing an increase in Aβ_1-40_ oligomers after oligomerization (*n* = 3, ***p < 0.001). (**C**) Light microscope images of ARPE-19 cells after exposure to Aβ_1-40_ oligomers (0.1 μM to 20 μM) for 24 h revealed no noticeable changes. (**D**) Light microscope images of ARPE-19 cells after exposure to Aβ_1-40_ oligomers (0.1 to 20 μM) for 48 h. There was a significant decrease in the cell number and change in cell shape in cells exposed to 20 mM Aβ_1-40_ oligomers. (**E**) Cell Counting Kit-8 (CCK-8) assay showed that the cell viability of ARPE-19 cells after the exposure to Aβ_1-40_ oligomers (0.1 μM to 20 μM) for 24 h did not change significantly (*n* = 4). (**F**) After exposure to Aβ_1-40_ oligomers for 48 h, ARPE-19 cell viability determined by CCK-8 assay was decreased after exposure to 20 μM Aβ_1-40_ oligomers for 48 h (*n* = 4, ***p < 0.001). Scale bar = 100 µm.

Aβ_1-40_ oligomers prepared from Aβ_1-40_ monomers were used as the in vitro AMD inducer in this study. The molecular weight of Aβ_1-40_ monomers was less than 10 KDa in the 0-h oligomerization group (Figure 1A). After 48 h of oligomerization, a smear of higher molecular weight proteins immunoreactive with the antibody 6E10 was observed, ranging approximately from 8 to more than 75 KDa. Semi-quantitative analyses also revealed a significantly increased level of Aβ_1-40_ oligomers after 48 h incubation (Figure 1B).

### 2.2. Aβ_1-40_ Oligomers (20 μM) Significantly Decreased Cell Viability after 48 h with Obvious Morphological Changes

Aβ_1-40_ oligomers-induced retinal pigment epithelium 19 (ARPE-19) cells were exposed to Aβ_1-40_ oligomers at different concentrations. After 24 h exposure, there was no noticeable morphological changes (Figure 1C) or cell viability loss (Figure 1E). However, after 48 h, cell morphology displayed remarkable changes. The cells shrank in size and became fragmented while surrounded by debris (Figure 1D). The cellular damage is also confirmed by Cell Counting Kit-8 (CCK-8) assay that cell viability was significantly decreased to 71 ± 3.1% after 48 h exposure to 20 μM Aβ_1-40_ oligomers (F = 59.187, *p* < 0.001) (Figure 1F). These results suggest that Aβ_1-40_ oligomers imposed significant toxicity on ARPE-19 cells at a concentration of 20 μM with 48 h-incubation.

### 2.3. LBP Exerted no Obvious Toxic Effects on ARPE-19 Cells from 3.5 to 14 mg/L

**Figure 2:**
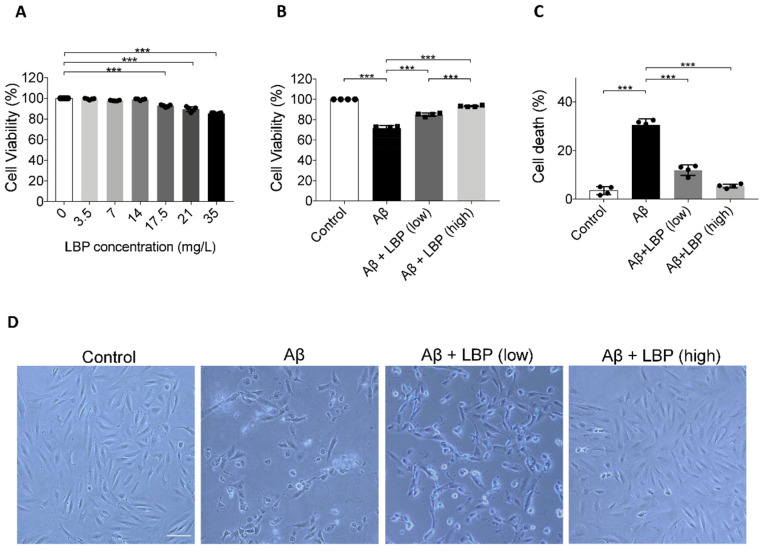
The effect of *Lycium barbarum* polysaccharides on ARPE-19 cell viability and morphology. (**A**) ARPE-19 cell viability after *Lycium barbarum* polysaccharides (LBP) treatment at various concentrations were determined by CCK-8 assay. No significant changes were observed for LBP treatment up to 14 mg/L. However, cell viability started to decrease when LBP was administered at 17.5 up to 35 mg/L. (*n* = 4, ***p < 0.001). (**B**) Exposure to Aβ_1-40_ oligomers decreased ARPE-19 cell viability (column 2). Administration of LBP at both low (3.5 mg/L) and high (14 mg/L) dosage were able to reverse the decreased cell viability imposed by Aβ_1-40_ oligomers (columns 3 and 4) (*n* = 4, ***p < 0.001). (**C**) Protective effects of LBP at low and high dosages were confirmed by trypan blue assay, which revealed that the ARPE-19 cell death rate was decreased by LBP treatment (*n* = 4, ***p < 0.001). (**D**) The morphological changes of ARPE-19 cells upon Aβ_1-40_ oligomers exposure with/without LBP treatment were examined by light microscopy. There was a decrease in cell number together with rounded cells and debris after Aβ_1-40_ oligomers exposure. The cells retained their normal morphology with LBP treatment (scale bar = 100 µm).

To evaluate the toxicity of LBP, ARPE-19 cells were exposed to LBP at different concentrations. CCK-8 assay results showed no statistical difference in ARPE-19 cell viability after exposure to LBP up to 14 mg/L for 24 h (Figure 2A). Yet, there was a significant decrease in cell viability (F = 14.837, *p* < 0.001) when LBP concentration reached 17.5 mg/L (Figure 2A). Based on these data, 3.5 mg/L (cell viability: 99.37 ± 0.71%) and 14 mg/L (cell viability: 98.88 ± 0.72%) were therefore chosen as the safe low and high LBP dose in the following experiments.

### 2.4. LBP Treatment for 24 h Improved Cell Morphology and Increased Cell Viability after Aβ_1-40_ Oligomers Exposure

To test the effect of LBP on the AMD cellular model, LBP was added to ARPE-19 cells exposed to Aβ_1-40_ oligomers. As shown previously, Aβ_1-40_ oligomers at 20 μM decreased cell viability (Figure 1F and Figure 2B). However, LBP at both low and high concentration rescued the reduced cell viability caused by Aβ_1-40_ oligomers (Figure 2B). Trypan blue assay also revealed that Aβ_1-40_ oligomers significantly increased the percentage of dead cells to 30.48 ± 2.57%, which was reversed by both low and high concentrations of LBP treatment, 11.94 ± 2.17% and 5.37 ± 0.82%, respectively (F = 163.90, *p* < 0.001) (Figure 2C). Moreover, the damaged cell morphology after Aβ_1-40_ oligomers exposure was also alleviated by LBP treatment for 24 h at both low and high concentrations (Figure 2D). These results suggested that LBP treatment was effective in reducing Aβ_1-40_ oligomers-induced ARPE-19 cell damages.

### 2.5. LBP Disrupts Aβ_1-40_ Oligomerization in a Dose-Dependent Manner

**Figure 3:**
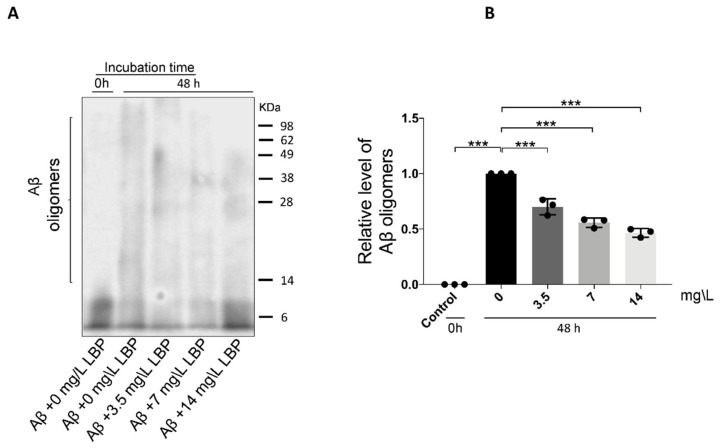
The effect of *Lycium barbarum* polysaccharides on Aβ_1-40_ oligomerization. (**A**) Representative Western blot illustrating the presence of Aβ_1-40_ monomers and oligomers with or without LBP incubation. The amount of the higher molecular weight Aβ_1-40_ oligomers generated after 48 h incubation decreased with increasing LBP concentration. (**B**) Semi-quantification of the relative level of Aβ_1-40_ oligomers based on the Western blot results (*n* = 3, ***p < 0.001).

To determine if LBP-mediated protection is associated with Aβ_1-40_ oligomers formation, LBP was added during the oligomerization process. As shown earlier, 48 h incubation generated Aβ_1-40_ oligomers ranging from approximately 8 to more than 75 KDa (Figure 1A and Figure 3A). With the addition of LBP during the oligomerization process, the amount of the higher molecular weight Aβ_1-40_ oligomers decreased (F = 76.91, *p* < 0.001) (Figure 3A). Semi-quantitative analyses revealed that the relative level of Aβ_1-40_ oligomers was significantly decreased with increasing concentration of added LBP (Figure 3B). These results suggested that LBP interrupted the process of Aβ_1-40_ oligomerization in a dose-dependent manner.

### 2.6. LBP Attenuated Aβ_1-40_ Oligomers-Induced Pyroptosis Pathway

**Figure 4:**
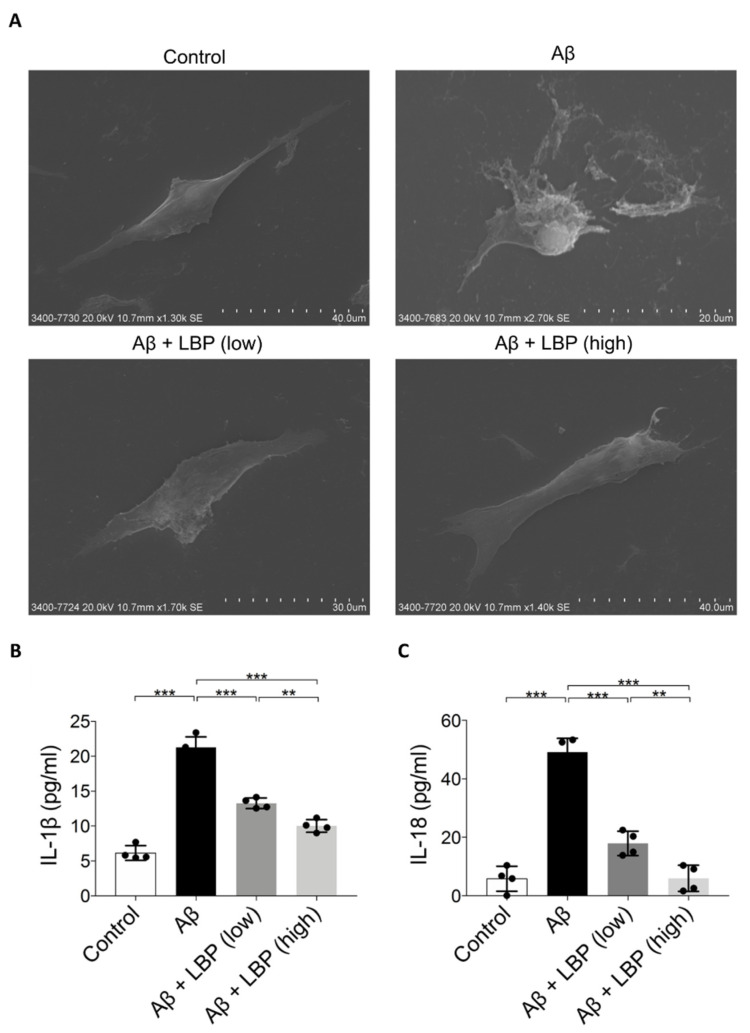
Aβ_1-40_ oligomers-induced cell death and release of pyroptotic products (IL-1β and IL-18) were alleviated by LBP treatment. (**A**). Scanning electron microscopy images revealed the morphology of ARPE-19 cells upon Aβ_1-40_ oligomers exposure with/without LBP treatment. Aβ_1-40_ oligomers exposure resulted in cell swelling and bubbling with a ruptured membrane while surrounded by membrane fragments and debris. LBP treatment recovered their normal morphology. (**B**). The concentration of IL-1β secreted by ARPE-19 cells was determined by ELISA. A significant increase of IL-1β in the cell culture supernatant was observed after Aβ_1-40_ oligomers exposure (column 2). Nevertheless, the level of IL-1β began to decrease when LBP was added (columns 3 and 4). LBP (low) represents a low concentration of LBP (3.5 mg/L) and LBP (high) stands for a high concentration of LBP (14 mg/L). (*n* = 4, ***p < 0.001). (**C**) IL-18 ELISA results showed that Aβ_1-40_ oligomers’ exposure resulted in a rise of IL-18 (column 2). However, LBP significantly reduced the level of IL-18 at both low (3.5 mg/L) and high (14 mg/L) concentration (columns 3 and 4). (*n* = 4, ***p < 0.001).

Light microscopy results revealed cell morphology changes after Aβ_1-40_ oligomers (Figure 2D). To further investigate the damage of Aβ_1-40_ oligomers imposed on RPE cell morphology, scanning electron microscopy images were obtained. Upon Aβ_1-40_ oligomers exposure, the ARPE-19 cell became swollen with a bubbling appearance and ruptured the cell membrane while surrounded by membrane fragments and debris. After LBP treatment, the cell shapes recovered, which was comparable to the control cells (Figure 4A). The morphological changes upon Aβ_1-40_ oligomers exposure were characteristics of the pyroptosis pathway [17,28]. Indeed, IL-1β and IL-18 levels as the final products of pyroptosis [29] were increased in ARPE-19 cells after Aβ_1-40_ oligomers exposure. These increases could be reduced by LBP treatment at both low and high concentrations (Figure 4B,C). In particular, IL-18 level in ARPE-19 cells could be reduced back to the level comparable to the control group with LBP treatment at the high concentration.

**Figure 5:**
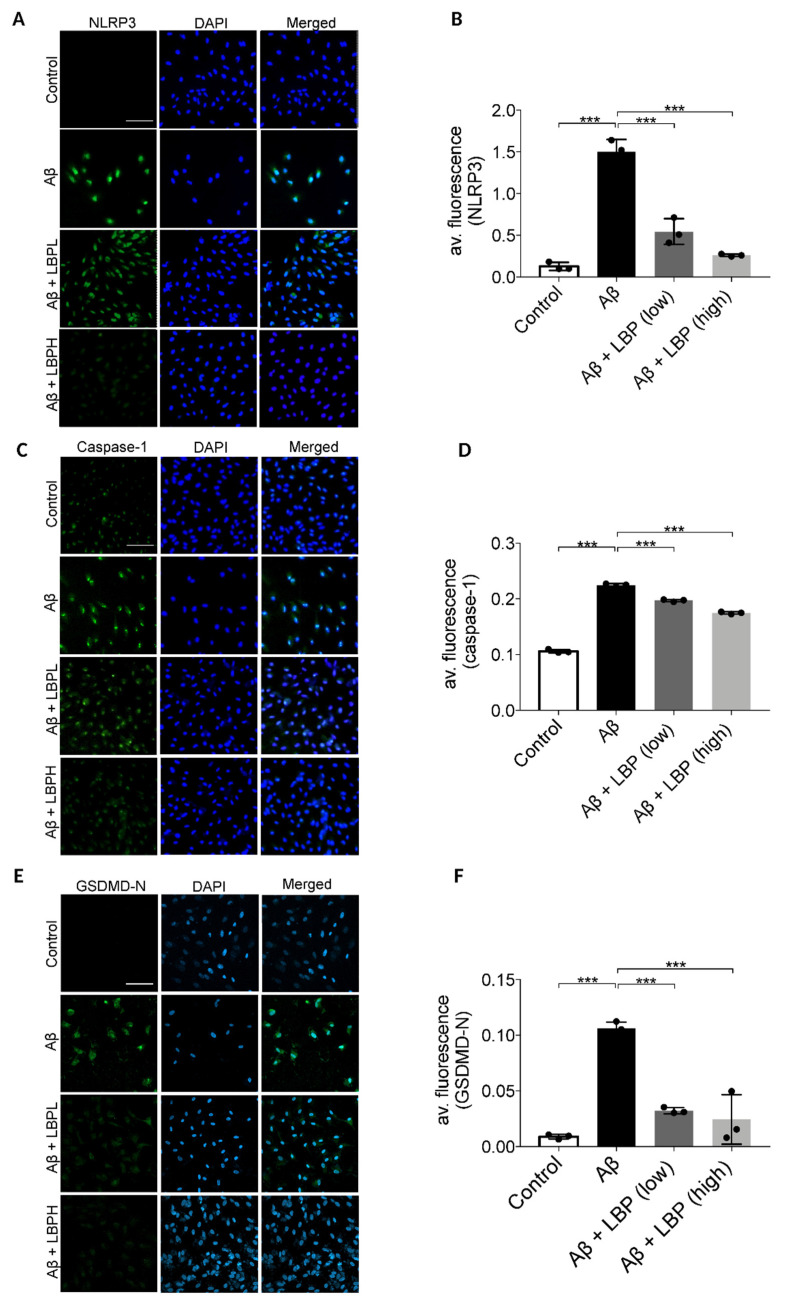
*Lycium barbarum* polysaccharides lessened the changes in expression of pyroptosis markers (NOD-like receptors protein 3 (NLRP3), caspase-1, and membrane N-terminal cleavage product of GSDMD (GSDMD-N)) in Aβ_1-40_ oligomers-exposed ARPE-19 cells. (**A**) Representative immunofluorescence images of NLRP3 (green fluorescence) and DAPI (blue fluorescence) in ARPE-19 cells under different treatments. Aβ_1-40_ oligomers exposure increased the cellular expressions of NLRP3 (second row). However, the increased expression was subsequently decreased by LBP treatment with both low (3.5 mg/L) and high (14 mg/L) concentration (third row and the fourth row). (**B**) The histogram indicated the average fluorescence intensity of NLRP3 based on the immunofluorescence results (*n* = 3, ***p < 0.001). (**C**) Representative immunofluorescence images of caspase-1 (green fluorescence) and DAPI (blue fluorescence) of ARPE-19 cells in different treatment groups. The expression of caspase-1 in ARPE-19 increased after Aβ_1-40_ oligomers exposure (second row). Nevertheless, the elevated expression was reduced by LBP treatment with both low (3.5 mg/L) and high (14 mg/L) concentration (third and fourth row). (**D**) The histogram for the average fluorescence intensity of caspase-1 based on the immunofluorescence data (*n* = 3, ***p < 0.001). (**E**) Representative immunofluorescence images showing the expression membrane GSDMD-N (green fluorescence) and DAPI (blue fluorescence) in ARPE-19 cells. There was a remarkable increase in GSDMD-N expression after Aβ_1-40_ oligomers exposure (second row). Nonetheless, LBP reduced the increased expression at both low (3.5 mg/L) and high (14 mg/L) concentration (third and fourth row). (**F**) The histogram for the average fluorescence intensity of GSDMD-N based on the immunofluorescence images (*n* = 3, ***p < 0.001). Scale bar = 100 µm.

To further confirm the involvement of pyroptosis, the expression of its various markers including NLRP3, caspase-1, and membrane GSDMD-N were examined by immunofluorescence assays. Aβ_1-40_ oligomers exposure for 48 h up-regulated cellular expressions of NLRP3 (second row) when compared with the control group (first row) (Figure 5A). The semi-quantitative analysis revealed that the average immunofluorescence intensity of NLRP3 was significantly increased in Aβ_1-40_ oligomers exposed cells (F = 96.689, *p* < 0.001) (Figure 5B). Aβ_1-40_ oligomers also increased the immunofluorescence signal (Figure 5C, second row) and the average immunofluorescence intensity of caspase-1 in ARPE-19 cells (F = 1133.310, *p* < 0.001) (Figure 5D). Furthermore, there was an increased expression of membrane GSDMD-N (second row) compared with the control group (first row) (F = 42.063, *p* < 0.001) (Figure 5E) with an increase of the average immunofluorescence intensity (Figure 5F). All these results demonstrated that Aβ_1-40_ activated the pyroptosis pathway in ARPE-19 cells. However, the increased expressions of NLRP3, caspase-1, and membrane GSDMD-N were subsequently down-regulated by LBP treatment at both low and high concentrations (Figure 5A–F), suggesting a protective role of LBP on the ARPE-19 cells in reducing pyroptosis upon Aβ_1-40_ oligomers exposure.

## 3. Discussion

**Figure 6:**
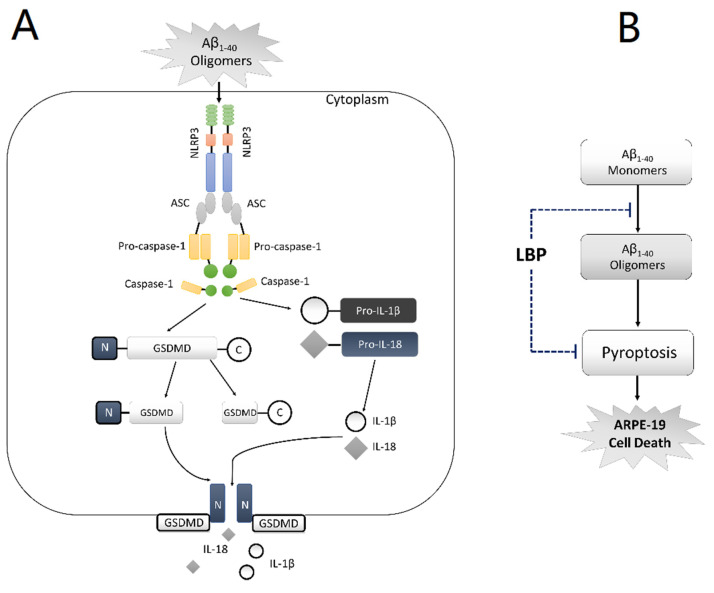
Schematic diagram of the proposed mechanism of amyloid β_1-40_ oligomer-induced ARPE-19 cell damage and LBP’s effects by anti-Aβ_1-40_ oligomerization and anti-pyroptosis. (**A**) In ARPE-19 cells, Aβ_1-40_ oligomers activate the NLRP3 inflammasome (NLRP3, the adaptor ASC, and pro-caspase-1), which subsequently leads to the generation of caspase-1. Caspase-1 cleaves Pro-IL-1β and Pro-IL-18, yielding IL-1β and IL-18, respectively. At the same time, C-GSDMD-N is also cleaved by caspase-1 thereby generating GSDMD-N and GSDMD-C. GSDMD-N moves to the cell membrane and forms ‘‘pores’’, allowing the newly generated IL-1β and IL-18 to be released from the cell. (**B**) *Lycium barbarum* polysaccharide (LBP) inhibits Aβ_1-40_ oligomerization and reverses the activation of pyroptosis, thereby protecting ARPE-19 cells from Aβ_1-40_ oligomers-induced cell death.

AMD is a complicated disease. The pathogenetic mechanisms are still not fully understood. Due to the limitation of the current therapeutic options, there is a need for novel effective treatment strategies. Goji berry has served as an effective Chinese herbal medicine for many eye diseases, but little is known about its effects in AMD. In the present study, our data suggested that pyroptosis is involved in RPE cell damage upon exposure to Aβ_1-40_ oligomers, which is a major constituent of drusen, and therefore possibly AMD development (Figure 6A). LBP treatment for 24 h significantly improved the cell morphology and cell viability by interrupting Aβ_1-40_ oligomerization and suppressing the pyroptosis pathway (Figure 6B). This study not only provides the first evidence on the involvement of pyroptosis in AMD development induced by Aβ_1-40_ oligomers and therefore a novel therapeutic target, but also contributes to the role of LBP as a potential new therapy for AMD.

To date, human RPE or ARPE-19 cells are used in in vitro AMD models with the frequently used inducers, Aβ_1-40_ [30], Aβ_1-42_ [31], hydrogen peroxide [32], or blue light [33]. Hydrogen peroxide or blue light are used for their ability to induce oxidative stress. Although oxidative stress plays an essential role in the pathogenesis of AMD [34], hydrogen peroxide and blue light cannot mimic drusen formation, which is the key pathology in the development of AMD and therefore these are not considered as the optimal choices for AMD in vitro studies.

Recently, a study using Aβ_1-42_ oligomers for in vitro AMD study reported that autophagy played a protective role in RPE cell survival [35]. However, the postmortem examination of AMD patients proved that Aβ_1-40_ is a major component of drusen, while Aβ_1-42_ was mostly regarded as an inducer of Alzheimer’s disease [8]; therefore, Aβ_1-40_ oligomers are considered a more proper inducer in in vitro AMD studies.

Two studies used Aβ_1-40_ oligomers, the key pathogenic component of drusen, in an in vitro AMD model on lipopolysaccharide (LPS)-primed ARPE-19 cells [30,36]. They suggested that the NLRP3 inflammasome and the oxidative stress pathways were activated by Aβ_1-40_ oligomers. Nevertheless, it is important to differentiate whether these activations were attributed to LPS or if Aβ_1-40_ oligomers can independently induce the activation. Another study showed that Aβ_1-40_ oligomers at 25 μM lowered ARPE-19 cell viability after 24 h of stimulation [10]. Nonetheless, the underlining mechanism, including the pathways of cell death or injury, remains to be further studied.

The current study provided novel evidence that the pyroptosis pathway was activated in non-LPS-primed ARPE-19 cells after Aβ_1-40_ oligomer exposure. Not only were NLRP3 and caspase-1 activated but GSDMD-N was also up-regulated, together with the characteristic morphological changes and an increased release of IL-1β and IL-18, which are the final products of pyroptosis. In fact, LBP can decrease the up-regulation of NLRP3, caspase-1, IL-1β, and IL-18 level in methionine choline-deficient diet steatohepatitis [37], hyperoxia-induced acute lung injury [38], and alcoholic cellular injury models [39]. This evidence supports a direct role of LBP in suppressing pyroptosis. A recent study showed that pyroptosis could also be induced in high glucose-treated ARPE-19 cells, resulting in an increased expression of NLRP3, caspase-1, Gasdermin D, IL-1β, and IL-18 [40]. It would be interesting to investigate if LBP is able to deliver a beneficial effect in these cells.

LBP is one of the major bioactive constituents isolated from Goji berry, with a wide spectrum of pharmacologic efficacy including neuroprotective [27,41,42], anti-inflammatory [43], anti-apoptotic [44], and immune-regulating effects [45]. The first study of Goji berry’s effects on macula was carried out in 2011. Bucheli et al. found that 90 days of Goji berry consumption in healthy elderly participants not only significantly increased antioxidant levels but also alleviated hypopigmentation and soft drusen accumulation in their macula [46]. Nevertheless, the therapeutic efficacy and the underlying mechanism of LBP on AMD is far from understood. This study is the first study to establish the ability of LBP in rescuing ARPE-19 cells from Aβ_1-40_ oligomers-induced toxicity via anti-Aβ_1-40_ oligomerization and the anti-pyroptosis pathway by down-regulating the increased expressions of NLRP3, caspase-1, and the membrane GSDMD-N as well as the release of IL-1β and IL-18.

## 4. Materials and Methods

### 4.1. Cell Culture and Chemical Treatment

The human adult retinal pigment epithelial cell line 19 (ARPE-19) was obtained from the ATCC (Homo sapiens, ATCC^®^ CRL2302 ™). Cells were cultured in DMEM/F-12 (Dulbecco’s Modified Eagle Medium/Nutrient Mixture F-12) (Gibco, cat no. 12400-016) supplemented with 10% fetal bovine serum (FBS), 100 U/mL penicillin, and 100 mg/mL streptomycin in humidified 5% CO_2_ at 37 °C. ARPE-19 cells at passage 9–15 were seeded in 6-, 12-, or 96-well plates and cultured in DMEM/F12 medium overnight at 37 °C. After starvation with serum-free medium for 24 h, the cells were treated with or without Aβ_1-40_ oligomers for 48 h. Subsequently, the Aβ_1-40_ oligomers containing-medium was removed. Then, LBP or vehicle (1×PBS) was added to these cells. After culturing with LBP-contained medium for 24 h, cells were harvested for different analysis.

### 4.2. Lycium barbarum polysaccharides (LBP) Preparation and Administration

*Lycium barbarum* polysaccharide (LBP), a gift from a Traditional Chinese Medicine manufacturer (Eu Yan Sang (Hong Kong) Ltd., Hong Kong), was prepared as previously described [25]. Briefly, *Lycium barbarum* sourced from Ningxia province of China was first boiled at 80 °C for 30 min and then soaked for 30 min. The resulting soup was subsequently concentrated by the soaking extracting method [47,48]. The technical bulletin stated that the final powder product contained a standardized 3.5% LBP while the remaining ingredients were mostly lactose.

For the experiments, the powder was freshly dissolved in 1×PBS and added to ARPE-19 cell culture medium at 100–1000 mg/L for 24 h to evaluate the safe dose. According to the standardized 3.5% LBP purity in the technical bulletin, the LBP dosage was therefore converted to 3.5–35 mg/L.

### 4.3. Aβ_1-40_ Oligomerization and Verification

The Aβ_1-40_ monomer (sequence: Asp-Ala-Glu-Phe-Arg-His-Asp-Ser-Gly-Tyr-Glu-Val-His-His-Gln-Lys-Leu-Val-Phe-Phe-Ala-Glu-Asp-Val-Gly-Ser-Asn-Lys-Gly-Ala-Ile-Ile-Gly-Leu-Met-Val-Gly-Gly-Val-Val) was synthesized by ChinaPeptide Ltd. company (catalog number: 04010011521, Shanghai, China) and reconstituted in 100% acetonitrile. After evaporation in the fume hood, a crystallized peptide was developed, which was then reconstituted in DMSO (5 mM) and aliquoted into 10 μL stocks. The aliquoted stocks were diluted in 1×PBS with 0.2% SDS to 400 μM (first dilution) and incubated for 18–24 h at 37 °C. The solution was further diluted with 1×PBS to 100 μM (second dilution), followed by incubation for another 18–24 h at 37 °C. The efficacy of Aβ_1-40_ oligomerization was verified by Western blot using 6E10 antibody (cat no. sig-39300, Biolegend, San Diego, CA, USA) as previously described [30].

### 4.4. Cell Counting Kit-8 (CCK-8) Assay and Imaging

To evaluate the cell viability of ARPE-19 cells exposed to Aβ_1-40_ oligomers, CCK-8 assay was performed using CCK-8 kit (cat no. CK-04-05. Dongjingdu, Japan). First, 1 × 10^4^ cells seeded in 96 well plates were treated with Aβ_1-40_ oligomers at different final concentrations (0, 0.1, 1, 5, 10, 15, and 20 μm) and incubated for 24 or 48 h. Afterwards, 10 μL CCK-8 liquid was added to each well and incubated for 1 h. Finally, absorbance was measured at 450 nm (BioTek, ELx800). Images of cell morphology was captured by a TE2000 inverted microscopic imaging system (Nikon, Tokyo, Japan).

### 4.5. Trypan Blue Assay

To assess the integrity of the cell membrane, trypan blue staining was performed. First, 5 × 10^4^ cells seeded in 12-well plates were treated with or without Aβ_1-40_ oligomers and LBP. After trypsinization for 1–2 min, a 10 μL cell suspension was mixed with 10 μL trypan blue (concentration: 0.4%, cat no. T6146, Sigma Aldrich) for less than 3 min. A hemocytometer was used to count the unstained (live) and stained cells (dead).

### 4.6. Incubation of Aβ_1-40_ Oligomerization with LBP

To study the effect of LBP on Aβ_1-40_ oligomerization, Aβ_1-40_ oligomerization was performed as described above until the second dilution. In the second dilution, 1×PBS was substituted with an equal volume of LBP at 3.5 or 14 mg/L, respectively, and incubated at 37 °C for another 18–24 h in 1.5 mL Eppendorf tubes. Western blot using 6E10 antibody was employed to detect the presence of oligomers from the tubes [27].

### 4.7. Immunoblotting

The Aβ_1-40_ monomers and oligomers were detected by Western blot assay. Eight μL and 100 μM Aβ_1-40_ from the second dilution (with or without performing oligomerization assay) were loaded into 12.5% SDS-PAGE and then transferred to polyvinylidene difluoride (PVDF) membranes. The membranes with blotted proteins were blocked with 5% non-fat milk and 0.001% Tween-20 in Tris-buffered saline (1×TBST) for 1 h and incubated with the primary antibody 6E10 (1:1000, cat no. Sig 393000. Biolegend, USA) at 4 °C overnight. It was washed with 1×TBST before incubation with the Rabbit Anti-mouse IgG H&L (Alexa Fluor^®^ 488) (1:500, cat no. ab150117. Abcam, CAM, United Kingdom) at room temperature for 1 h. An Amersham Imager 680 imager was used to visualize the blots bands. The captured images were analyzed by ImageJ. Briefly, using the image generated from an Amersham Imager 680, a rectangular area around the bands of interest was selected. Since the molecular weight of the Aβ_1-40_ monomer is about 4 KDa, the lower limit of the rectangle was set at 4 KDa. As the highest molecular weight of the Aβ_1-40_ oligomers could not be determined, the upper limit of the rectangle was set at the edge of the separating gel. The Western blot and ImageJ analyses were performed at least three times.

### 4.8. Scanning Electron Microscopy

To investigate the morphological changes in Aβ_1-40_ oligomers-induced ARPE-19 cell damage, scanning electron microscopy was performed on ARPE-19 cells seeded in 12-well plates (5 × 10^4^ cells/well) and treated with or without Aβ_1-40_ oligomers or LBP. Then, cells were fixed with 4% paraformaldehyde for 40 min and washed with 1×PBS 4 times. The samples were dehydrated in a graded series of ethanol and dried with the critical point dryer. The sample surface was coated with a thin layer of gold-palladium film and visualized with a Hitachi S-3400N variable pressure scanning electron microscope (Electron Microscopy Unit, Queen Mary Hospital, The University of Hong Kong).

### 4.9. Enzyme-Linked Immunosorbent (ELISA) Assay

To assay for pyroptosis products, ELISA was performed. In short, 5 × 10^4^ cells seeded in 12-well plates were treated with or without Aβ_1-40_ oligomers or LBP. Subsequently, cell culture supernatant was collected. IL-1β and IL-18 levels in the cell culture supernatant were measured with a commercial Human IL-1 β ELISA Kit (Abcam, ab46052) and Human IL-18 ELISA Kit (Abcam, ab215539), respectively. The optical density (OD) values at 450 nm were measured by an absorbance reader (BioTek, ELx800, LabX, Midland, ON, Canada). The level of IL-1β and IL-18 in the cell culture supernatant was estimated using a standard curve as recommended by the manufacturers. All samples were assayed in duplicate.

### 4.10. Immunofluorescence Assay

To study the expression of pyroptosis markers, immunofluorescence assays were employed. Briefly, ARPE-19 cells were seeded in 12-well plates (5 × 10^4^ cells/well) and cultured overnight. After serum-free medium starvation, the cells were exposed to Aβ_1-40_ oligomers for 48 h, followed by LBP treatment for 24 h. Then, the cells were fixed with 4% paraformaldehyde and permeated with 0.1% Triton X-100. Triton X-100 was excluded for the detection of membrane GSDMD-N. After blocking with 3% bovine serum albumin, cells were subsequently incubated with primary antibodies at 4 °C overnight, followed by incubation with the secondary antibody for 1 h at room temperature. Nuclei were stained with DAPI and mounted with mounting medium. Fluorescence images were captured by a TE2000 inverted microscopic imaging system (Nikon, Tokyo, Japan).

Immunofluorescence intensity was analyzed using ImageJ following previously published protocols [49,50]. The average fluorescent intensity was calculated by dividing the corrected optical density by total area of fluorescence. The immunofluorescence assay and Image J analysis were performed at least three times.

The primary antibodies used for immunofluorescence were as follows: NLRP3 (1:500, cat no. NBP2-12446. Novus biologicals, CO, USA), Caspase-1 (1:500, cat no. sc-392736. Santa Cruz, CA, USA), and GSDMDC1 (1:500, cat no. sc-81868. Santa Cruz, CA, USA). Secondary antibodies were goat anti-rabbit IgG H&L (Alexa Fluor^®^ 488) (1:500, cat no. ab150077. Abcam, CAM, United Kingdom) and rabbit anti-mouse IgG H&L (Alexa Fluor^®^ 488) (1:500, cat no. ab150117. Abcam, CAM, United Kingdom).

### 4.11. Statistical Analyses

All experiments were performed in duplicate and repeated at least three times independently. Data were shown as mean ± standard deviation and analyzed by one-way ANOVA followed by Bonferroni’s post hoc test (GraphPad Prism^®^ 7 software). A *p*-value < 0.05 was considered statistically significant.

## 5. Conclusions

To conclude, our results demonstrated that the exposure of Aβ_1-40_ oligomers resulted in the activation of pyroptosis in ARPE-19 cells. More importantly, LBP can rescue the Aβ_1-40_ oligomers induced-RPE cell damage by anti-oligomerization and anti-pyroptosis, indicating its potential as a therapeutic agent for the treatment of AMD.

Despite demonstrating the beneficial effects of LBP in disrupting Aβ_1-40_ oligomerization in vitro while protecting ARPE-19 cells upon Aβ_1-40_ oligomers exposure, the cellular model is not sufficient to mimic the in vivo AMD pathological processes. Thus, further in vivo studies are warranted.
